# Assessment of predictors for acute asthma attack in asthmatic patients visiting an Ethiopian hospital: are the potential factors still a threat?

**DOI:** 10.1186/s40733-018-0044-7

**Published:** 2018-07-16

**Authors:** Sewunet Admasu Belachew, Daniel Asfaw Erku, Dawit Kumilachew Yimenu, Begashaw Melaku Gebresillassie

**Affiliations:** 1 Department of clinical pharmacy, School of Pharmacy, University of Gondar, P.O. Box: 196, Gondar, Ethiopia; 2 Department of pharmaceutics and social pharmacy, School of Pharmacy, College of medicine and health sciences, University of Gondar, Gondar, Ethiopia

**Keywords:** Asthma exacerbation, Ethiopia, Gondar, Hospital, Factors

## Abstract

**Background:**

Recurrent exacerbations in patients with moderate or severe asthma are the major causes of morbidity, mortality and medical expenditure. Identifying predictors of frequent asthma attack might offer the fertile ground of asthma management. However, systematic data on asthma management is scarce in Ethiopia.

**Objective:**

The purpose of the present study was to determine predictors of acute asthma attack in patients with asthma attending emergency department of University of Gondar Comprehensive Specialized Hospital (UOGCSH) in Gondar, northwestern Ethiopia.

**Methods:**

An institutional-based cross-sectional self-administered survey was conducted on 108 asthmatic patients who came to the emergency department of UOGCSH following acute asthma attack. Data were collected through interviewer administered questionnaire. Logistic regression was done to see the possible association of potential factors that may lead to asthma exacerbation.

**Result:**

About half of the respondents (51.9%) were female and one third of patients (38.9%) were within the age range of between 46 and 60 years. The leading potential predictor were frequent exposure to various ongoing allergen (68.5%) followed by revelation to occupational sensitizers (67.6%). Chronic sinusitis (AOR = 3.532, 95% CL = 1.116–11.178), obstructive sleep apnea (AOR = 3.425, 95% CL = 1.255–9.356) and psychological disfunctioning (3.689 (1.327–10.255)) were among the significantly associated factors of acute asthma exacerbation.

**Conclusions:**

Now days, the backbone for long-term asthma management is to prevent exacerbations. Chronic sinusitis, obstructed sleep apnea and psychosocial dysfunction were originated to be considerably linked with repeated exacerbations of asthma. Among those significantly associated predictors, obstructed sleep apnea were the most prevalent one.

## Background

Asthma is a universal health obstacle jeopardizing beyond 300 million individuals’ life of all ages, ethnic groups, and countries [1]. However, due to ecological variation, there is a considerable heterogeneity of asthma regarding gene-environment interactions, environmental acquaintances, comorbidities, age, causal disease harshness, health care availability, psychological factors, disease response to management, and disease load including asthma exacerbations and mortality as well as long-term chronic morbidity [2, 3]. Although asthma is most common in developed (westernized) countries, it is alarmingly growing in developing countries due to the amplified urbanization of communities [4, 5]. Acute asthma attacks remain a cause for regular encounter to emergency department in a hospital as a new admissions [6]. Overall, acute asthma episodes- especially hospital encounters account for uneven health expenditures compared with the treatment of stable asthma [7]. Acute exacerbation events usually give time for intervention; however in some occasions the onset of the symptoms become so rapid, better to be distinguished from periods of deprived asthma control [8–10]. However, some patients hurt from frequent asthma attack resulting in: working days and school absence, frequent emergency admission. Such asthma attacks are linked with extensive injury and constitute a significant percentage of the gross expenditures of asthma [11–13]. Emergency unit and hospitalization for asthma remain to be related to several psychosocial factors, such as inferior socioeconomic status inaccessibility of medical care and already standing mental disorders [1]. Moreover, patient individualities that drives to lethal asthma exacerbations include being female, elderly, tobacco user and non-adherence to treatment [4, 10, 11]. In addition, several modifiable and non-modifiable precipitating factors were identified in complicated asthma [14–16]. However, it is mysterious that which factors leads to the recurrence of acute asthma attack in patients with poorly controlled disease. Despite the clinical and economic prominence of acute asthma attack, merely a few researches have surveyed predictors that may lead to disease recurrence. Therefore, this study will help fill this gap and identify those predictors for unsuccessful Asthma control as well as Asthma exacerbations. This will help in the enactment of early therapeutic interventions aimed at correcting these factors which are expected to reduce morbidity and medical expenditure.

The study will also support the goverment in the effort to step down expenses for purchasing medications and by saving lost work days following Asthma attacks as well as subsequent hospital admissions. This study has also an importance for upcoming studies to be used as reference. With this, the purpose the present study is to identify predictors of acute asthma exacerbation among patients with asthma who came to emergency department of UOGCSH, Gondar town, Northwestern Ethiopia.

## Methods

### Study area and design

An institutional based cross sectional survey was done on adult patients who came to the emergency medicine unit of UOGCSH following asthma exacerbations. UOGCSH is the oldest referral hospital located northwestern Ethiopia. The Hospital has many wards, comprised of; Internal medicine, gynecology/obstetrics, pediatrics, surgery units and one big emergency unit. The study period was from January 1 to May 10, 2017.

### Sampling

A Convenience sample of all patients with asthma who visited the hospital following acute asthma attack during the study period were included. An overall of 115 patients were invited. Patients who were capable of understanding the questionnaire were included, while patients with serious psychological/physical disorder and who rejected an offer to participate in the study were excluded. With this our study participants reduced to a final sample of 108 patients.

### Data collection and management

The tool used was adopted from the previously peer-reviewed studies after brief modifications. The final format has two major parts: The first part: - demographic items (age, sex…) while the second part included items related potential predictors and adherence to asthma therapy. The data were collected by four well-trained Clinical Pharmacists through interviewer-administered questionnaires. The tool, originally written in English, was translated to local language (Amharic) then translated back to English by anther expert in order to ensure that the translated version gives the original meaning. To clarify, the diagnosis of the possible asthma exacerbating diseases such as Obstructive sleep apnea,chronic sinusitis,psychological dysfunctions and the like were made by either internship medical students, senior physicians/nurses or health officers right away at UOGCSH when they came for the management of the acute asthma attack or it was identified as the diagnosis had been already made in other else local health centers by the same professionals and in the latter case, the patient lived for a longer time with the mentioned asthma exacerbating disease but the patient failed to control it . In both occasions, as to the explanation of the respondents and the data collectors as an eye witness at UOGCSH, the diagnosis were made based on brief patient history and physical examination. The content validity of the tool was revised and reassured by a group of experts, including Internists physicians, health information experts, and clinical pharmacists. Pretest was employed on 20 asthmatic patients prior to the gross data collection, which was left out from the final study, and pertinent amendments were applied.

### Statistical analysis

The final data collection tool were carefully checked for completeness and the filled responses first entered to and analyzed using the Statistical Package for the Social Sciences software Version 20 for Windows. The demographics of study subjects were clearly described using frequencies, percentages, tables, figures and logistic regression were used to illustrate and to explore potential associations of different variables and findings. *P-*value of 0.05 with CI of 95% was considered as a cut-off point to declare significance.

### Ethical consideration

This research was ethically approved by the Ethical Review Committee of School of Pharmacy, University of Gondar with an approval number of University of Gondar (UoG)-School of Pharmacy (SoP)-120–2017. Permission from the emergency department of the hospital was also received. Written informed consent from participants was also gained prior to conducting the study. Participants’ information obtained from the questionnaires was kept confidential via data coding. Participants were also informed that participation was voluntarily.

## Result

### Patients’ demographic characteristics

For the 108 chronic patients with asthma who involved in the study; factors for asthma exacerbation and the prevalence of those potential contributing factors was assessed. About half of the study subjects (51.9%) were female and one third of patients (38.9%) were within the age range of between 46 and 60 years with a median age of 48 years. 65.7% of the respondents were urban residents and almost half of the total sampled patients (55.6%) and (50.0%) know only little about their disease and their medications, respectively. In line with this, about 23.1 and 30.6% of patients doesn’t know anything about their disease and medications, respectively. The median asthma duration was 13 years with one fourth of patients (25.9%) having duration of between 5 and 10 years. The detailed characteristics of study participants are shown in [Table 1].

**Table 1:** Socio-demographic characteristics of the study participants

Variables	Frequency, N (%)
Gender	
Male	52 (48.1)
Female	56 (51.9)
Age group	
18–25	14 (13)
26–35	19 (17.6)
36–45	19 (17.6)
46–60	42 (38.9)
> 60	14 (13)
Marital status	
Unmarried	27 (25)
Married	69 (63.9)
Divorced	2 (1.9)
Widowed	10 (9.3)
Educational status	
No formal education	41 (38)
Secondary education	17 (15.7)
Primary education	25 (23.1)
Tertiary level	25 (23.1)
Employment status	
Unemployed	58 (53.7)
Employed	42 (38.9)
Retired	2 (1.9)
Student	6 (5.6)
Monthly income	
< 500	5 (4.6)
500–1500	75 (69.4)
1501–2500	17 (15.7)
> 2500	11 (10.2)
Area of residency	
Rural	37 (34.3)
Urban	71 (65.7)
Family history of asthma	
Yes	27 (25.0)
No	81 (75.0)
Duration of asthma	
< 5 years	31 (28.7)
6–10 years	28 (25.9)
11–15 years	15 (13.9)
16–20 years	15 (13.9)
> 20 years	19 (17.6)
Knowledge about asthma	
Very good	23 (21.3)
Little	60 (55.6)
No knowledge	25 (23.1)
Knowledge about asthma medications	
Very good	21 (19.4)
Little	54(50.0)
No knowledge	33 (30.6)

**Fig. 1:**
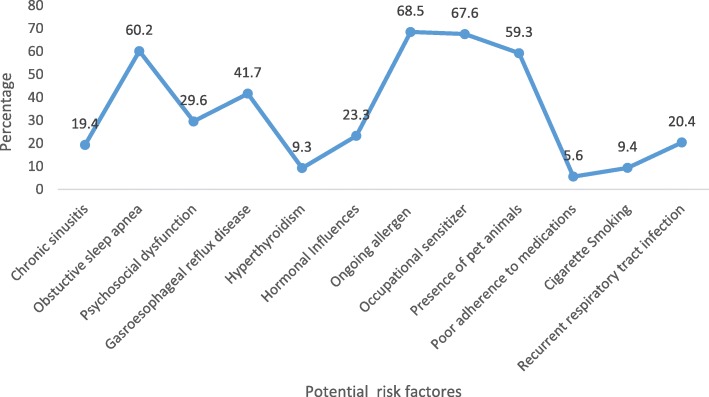
Prevalence of potential risk factors of frequent asthma attack in total study population included in the analysis

In the study, many potential predictors of asthma exacerbation were identified, with this the leading one was frequent exposure to several ongoing allergen (68.5%) followed by contact to work-related sensitizers (67.6%) [Fig. 1].

### Factors associated with frequent asthma exacerbations

Appropriate analysis model was instituted to determine predictors of frequent severe exacerbations for each of the variables in the eq.

A large number of potential factors found to be significantly associated with a minimum of one asthma exacerbation within the last two years in a bivariate analysis [Table 2]. Among factors analyzed for significant association recurrent respiratory tract infections (OR 2.953, CI 95%), chronic sinusitis (OR 3.437, CI 95%), obstructive sleep apnea (OR 3.394, CI 95%), gastro-esophageal reflux disease (OR 3.474, CI 95%), psychological dysfunction (OR 3.205, CI 95%), ongoing allergen exposure (OR 2.778, CI 95%), exposure to occupational sensitizers (OR 2.969, CI 95%) were found to be significantly associated with acute asthma attack. Other factors with potential association but not significant were; Present use of salicylates, NSAIDS, B-blockers, ACE inhibitors (OR 1.671, CI 95%), poor adherence to medication (OR 1.412, CI 95%), presence of pet animals at home (OR 1.123, CI 95%), urban residency (OR 1.455, CI 95). The details of the potential factors associated with frequent asthma exacerbations as to the result of crudes odds ratio are described below at [Table 2].

**Table 2:** Crude odds ratio (COR) for factors associated with frequent asthma exacerbation

Factors	COR (95% CI)	*P* value
Recurrent respiratory tract infections	2.953 (1.117–7.810)	0.029
Presence of chronic sinusitis	3.437 (1.256–9.405)	0.016
Presence/ history of obstructive sleep apnea	3.394 (1.464–7.869)	0.004
Presence of gastro esophageal reflux disease	3.474 (1.556–7.756)	0.002
Presence of psychological dysfunctioning	3.205 (1.359–7.562)	0.008
Ongoing allergen exposure	2.778 (1.143–6.749)	0.024
Exposure to occupational sensitizers	2.969(1.224–7.202)	0.016
use of salicylates, NSAIDS, B-blockers, ACE inhibitors	1.671 (0.721–3.872)	0.231
Adherence		
Very good	–	0.043
Good	3.840 (0.657–2.429)	0.135
Poor	1.412 (0.222–8.990)	0.715
Presence of pet animals at home	1.123 (0.516–2.446	0.769
Residency	1.455 (0.653–3.242)	0.359

After adjusting for potential confounders we tried to put the adjusted odds ratio and we come with a result that only chronic sinusitis (adjusted OR (95% CI) 3.532(1.116–11.178)), presence of obstructive sleep apnea (adjusted OR (95% CI) 3.426 (1.255–9.356)), presence of psychological dysfunctioning (adjusted OR (95%) 3.689 (1.327–10.255)) were the mere factors found to be significantly associated with frequent asthma exacerbations. The details of the adjusted odds ratio resulted described below at [Table 3].

**Table 3:** Adjusted odds ratio (AOR) for factors associated with frequent asthma exacerbations

Factors	AOR (95% CI)	*P* value
Infections	1.534 (0.470–5.013)	0.479
Sinus	3.532 (1.116–11.178)	0.032^*^
Sleep apnea	3.426 (1.255–9.356)	0.016^*^
GERD	1.865 (0.703–4.943	0.210
Psychological disfunctioning	3.689 (1.327–10.255)	0.012^*^
Allergen exposure	1.558 (0.495–4.900)	0.448
Occupational sensitizers	2.044 (0.677–6.176)	0.205

*significantly associated at *p* < 0.05

## Discussion

This original article was aimed to pinpoint the factors associated with acute asthma exacerbations in asthmatic patients. Different scientific outputs disclosed that occurrence of asthma attack have been frequent among asthmatic patients and it is highly linked with various potential factors such as: Sinus diseases, Gastro esophageal reflux disease, and mental problem [17].

Among 108 study participants, beyond half of them have little knowledge about their disease condition and medications they are taking, (60)55.6% and (50)54%, respectively. This might be due to their education status since huge number of patients 41(38%) were not in formal education and majority of patients were very older (46-65 years) so that they could not be energetic to update themselves about their medical therapy and their disease status. This results concurs with the study done in Europe which claims that there were patients’ lack of knowledge about asthma and its treatments which is referred as main obstacle among adult asthmatics and can estimate poor asthma control [18]. The duration of asthma per year (5-10 years) was comparable with the study done in Canada [10].

The study also tried to discover different potential predictors of asthma poor control and asthma attack and come up with factors like sinus disease, obstructive sleep apnea, Gastro esophageal reflux disease, psychological dysfunctions, smoking, and exposure to allergen and another number of elements. Among the potential predictors explored, the leading one was frequent exposure to several ongoing allergen(68.5%) followed by exposure to work-related sensitizers(67.6%).This could be justified as huge number of patients live in urban area which is believed as the key birthplaces of enormous allergens and occupational sensitizers compared to rural areas since those places are usually preferential locations for industries and other potential factories which are all widely known to emit lots of allergens and sensitizers. The possible mechanism of inhaled allergens including occupational sensitizers to cause acute asthma attack is via activating mast cells that render them to release histamines, leukotriene, Interleukin and prostaglandins that in turn lead to bronchospasm that further increase airway responsiveness as well as airway limitations. Moreover, those mediators end up with infiltrations of inflammatory cells like cytokines and other inflammatory mediators that results to airway inflammation characterized by edema, epithelial injury and impaired mucocillary functions [19, 20].

The study finding was incomparable with the study done in Netherland in which the front line factor identified was presence of recurrent respiratory tract infection followed by Gastro esophageal reflux disease which is quite different from our study result [17]. Having this in mind, statistical analysis was done to point out the probable is a statistical significances between those potential predictors and incidence of frequent asthma attacks. The multivariate analysis showed that Asthmatic patients having chronic sinusitis were 2.5 times more like to encounter acute asthma attack compared to those who have not yet faced this co-morbid (AOR = 3.532, 95% CL = 1.116–11.178).This might be due to the fact that large number of the study subjects live in urban area and in this place there are many chemicals, gases and the like that will worsen the sinusitis status then end up with acute attack. Both seasonal and viral chronic sinusitis are among the most common triggers of acute severe exacerbations and may invade epithelial cells of the lower as well as the upper airways and there is an increase in airway inflammation with increased numbers of eosinophil and neutrophils along with nasal congestion as well as airway tract edema in addition to increment in airway hyper-responsiveness [19, 20]. The existence of obstructive sleep apnea(OSA) also was 2.4 times more like to run in to acute asthma attack compared to those who had not (AOR = 3.425, 95% CL = 1.255–9.356). The possible mechanisms by which OSA lead to the worsening of asthma control in patients with concomitant OSA include Neuro-mechanical reflex bronchoconstriction because of increase vagal tone while sleeping, increased prevalence of gastro-esophageal reflux with OSA, increased inflammation and the indirect effect on dyspnea of OSA-induced cardiac dysfunction [21]. In addition to those, owing psychological dysfunction was 2.6times more like to run in to acute asthma attack compared to those who had not (AOR = 3.689,95%CL = 1.327–10.255). Basically, in Psychological dysfunctioning, the probable mechanism to acute worsening of asthma have been related with stress. There is no doubt that psychological factors can induce bronchoconstriction through cholinergic reflex pathways [19]. Those findings were comparable with the study done in Netherland, Leiden University (AOR =3.7, 95 CL = 1.2–11..9,AOR = 3.4, 95% CL = 1.2–10.4,AOR = 10.8,95%CL = 1.1–108.4, respectively) [17].

Apart from those factors, the multivariate analysis showed that other potential predictors were not significantly associated with asthma exacerbation but to the contrary the study conducted in Netherland claimed that factors like recurrent respiratory tract infections(AOR = 6.9,95%CL = 1.9–24.7), Gastro-esophageal reflux (AOR = 4.9,95%CL = 1.4–17.8) are significantly associated with acute asthma exacerbations [16]. In GERD, acid reflux might trigger reflex bronchoconstriction bur rarely cause asthma symptoms [19]. Unlike this study, one study revealed that pathological gastro-esophageal reflux is considered a potential trigger of asthma, even in the non-existence of esophageal symptoms [22]. To strengthen the point rose regarding chronic sinusitis which had been suggested to take part in poor controlled asthma and asthma exacerbation.

Appropriate treatment of sinusitis in asthmatic patients have been shown to result in both improved Sino nasal and asthmatic symptoms with very fewer physician visits and decreased need for medication in several patients [23, 24]. Comparably to our study, lots of psychosocial factors have been drivers of severe frequent asthma attacks [17, 22]. A study stated that, if psychological disturbances are recognized and properly managed, frequency of acute asthma attack and the detrimental consequences might be significantly abridged [23].

In line with the above mentioned predicators, there are also other factors that had been identified to be associated with acute asthma attack although were proved to be not significant. Those were medication used concomitantly such as salicylates, ACE inhibitors, B-blockers and the like. To mention the probable mechanisms, Angiotensin-converting enzyme inhibitors are theoretically detrimental as they inhibit breakdown of kinins, which are broncho-constrictors while Aspirin augments the leukotriene path that lead bronco constrictions. In addition, although the mechanisms of beta-blockers are not clear but are likely mediated through increased cholinergic bronchoconstriction [19].

As a limitation, the present study was done in a single town and cannot be fully generalized to other places in Ethiopia although the result can be used as input for upcoming nationwide investigations. In addition, all measures used were based on self-reporting, this might end up with socially desirable responses.

## Conclusion

Now days, the backbone for chronic asthma management is to prevent its exacerbations. This calls for improved understanding of predictors that contribute to the incidence of severe disease exacerbations. The present study, looking for asthmatic patients according to a systematic protocol, provides a clinical profile of the patients with acute asthma attack. In particular, chronic sinusitis, obstructive sleep apnea and psychosocial dysfunction were found to be significantly associated with frequent acute asthma attack. Among those significantly associated predictors, Obstructive sleep apnea were the top one.

### Implications

It can be anticipated that rigorous treatment of these comorbid factors will end up in less asthma exacerbations with better disease control, which will prominently enhance the quality of life of patients with chronic asthma. Taking into consideration the heightened importance of clearly identifying the reasons for repeated hospital admissions following acute asthma exacerbations so as to prevent the number of chronic asthmatics who encounter acute exacerbations, different stakeholders working in the health sector particularly in asthma control should provide a customized health promotion intervention and awareness creation to chronic asthmatic patients living in urban and rural areas. Finally, taking the result of this study as input, we do recommend researchers to work on a multi-centered study with a huge number of sample population for better generalization.
